# Obstetric and Gynecological Admissions and Hospitalizations in an Italian Tertiary-Care Hospital during COVID-19 Pandemic: A Retrospective Analysis According to Restrictive Measures

**DOI:** 10.3390/jcm12227097

**Published:** 2023-11-15

**Authors:** Gaetano Riemma, Pasquale De Franciscis, Marina Tesorone, Egle Coppa, Antonio Schiattarella, Valentina Billone, Alessandra Lopez, Gaspare Cucinella, Giuseppe Gullo, Raffaela Maria Carotenuto

**Affiliations:** 1 Department of Woman, Child and General and Specialized Surgery, University of Campania “Luigi Vanvitelli”, 80138 Naples, Italy; pasquale.defranciscis@unicampania.it (P.D.F.); egle.coppa@studenti.unicampania.it (E.C.); aschiattarella@gmail.com (A.S.); raffaelamaria.carotenuto@studenti.unicampania.it (R.M.C.); 2 Local Health Services “Napoli 1 Centro”, 80138 Naples, Italy; marina.tesorone@aslnapoli1centro.it; 3 Department of Obstetrics and Gynecology, Villa Sofia Cervello Hospital, University of Palermo, 90146 Palermo, Italy; valentinabillone@gmail.com (V.B.); alessandralopez91@gmail.com (A.L.); gaspare.cucinella1@gmail.com (G.C.); gullogiuseppe@libero.it (G.G.)

**Keywords:** COVID-19, hospital admissions, emergency care, obstetric urgency, lockdown, restrictions

## Abstract

Background: The national lockdown and the different restrictions applied in 2020 during the COVID-19 pandemic brought several changes to hospitalization procedures. The aim of this study was to evaluate the patterns in access to emergency services and hospitalization in a tertiary-care obstetric and gynecological emergency department (OG-ED) throughout the restrictions applied during 2020. Methods: A single-center retrospective comparative study on data from January to December 2020 was carried out on the following timeframes: January to February 2020 (before COVID-19 pandemic), March to June 2020 (nationwide lockdown period), July to September 2020 (removal of restrictive measures), October to December 2020 (regional lockdown) and compared to the same periods of 2019. All obstetric and gynecological patients with complete medical data admitted to the OG-ED were included. Results: Overall, 4233 accesses for 2019 and 3652 for 2020 were reported, with a decreasing trend of −13.7%. Between March and June 2020 (nationwide lockdown) and 2019, the overall number of patients attending the OG-ED decreased compared to July–September and October–December differences (Δ −23.5% vs. −3.1% and −5.9%; *p* = 0.001 respectively) for 2020–2019, but this reduction was not statistically significant when compared to January–February (Δ −23.5% vs. −18.5%; *p* = 0.356). No significant differences for obstetric patients (Δ −1.8% vs. −1.0% vs. −2.3% and +1.9% respectively; *p* = 0.883) were noted. Hospitalizations showed a stable trend with an increase between October–December 2019 and 2020 (Δ +4.6%; *p* = 0.001 vs. January–February (+2.4%) and March–June (+2.6%) 2019–2020), mainly related to regional lockdowns. Conclusions: In contrast to available national studies, in our institution, the overall rate of OG-ED admissions was slightly reduced with a similar trend of decrease even before COVID-19, with an increase in admissions for serious issues, despite expectations that the suspension of elective admissions and outpatient services would have led to an increase in non-urgent hospitalizations during the COVID-19 lockdown period.

## 1. Introduction

On 11 March 2020, the World Health Organization (WHO) released its 51st status report, in which the SARS-CoV-2 virus outbreak, also known as COVID-19, was classified as a pandemic [1]. Over the year 2020, to enforce social isolation and stop the spread of the SARS-CoV-2 virus, the Italian government implemented several restrictions [2].

Such restrictions were repeated in different periods according to each wave that affected Italy. In particular, the most severe lockdown was related to the wildtype SARS-CoV-2 variant and took place between March 9th and June 2020, in which a state of emergency, also known as “lockdown”, was applied [3]. All non-essential activities, including office labor, commerce, sports, and leisure pursuits, as well as unrestricted movement inside and outside of the nation, were outlawed. Citizens were asked to not leave their home except for emergency reasons (e.g., urgent care needs) [3].

With the reduction in the number of infected people, such restrictions were gradually loosened to the use of masks in crowded areas between July and September 2020 [4,5]. However, with the second COVID-19 wave that took place from October 2020, with an exponential increase in infected people, Italian regions were grouped into three different epidemiological scenarios (known as yellow, orange, and red zones), with a nationwide curfew from 10 p.m. to 5 a.m., shopping malls were ordered to be closed on weekends, and distance education was used for high schools [4]. For regions in “orange zones”, a ban on travel outside the municipality of residence and the closure of food services were extended, while for regions in “red zones”, a ban on travel even within the municipality, the closure of stores and markets, and the use of distance education from seventh grade onward were applied [4].

During and after the lockdown, Italian hospitals significantly changed their routine by postponing all non-urgent outpatient visits and planned operations [6]. All urgent and emergent needs, including mandatory outpatient check-ups, were assured. This was carried out to relieve pressure on the intensive care units (ICUs) of hospitals that served COVID-19-positive patients and to save costs [6]. During the pandemic, ICU accessibility was crucial and lifesaving [7].

Consequently, after the initial SARS-CoV-2 infection spread to Italy, there was an abrupt and significant decline in emergency department visits. Overall, emergency department attendance drastically dropped by 41.8% from the previous year starting on 21 February [8]. Both medical and surgical wards were changed to COVID-19 units, and new supplemental sub-intensive and ICUs were established for COVID-19 patients. Elective surgery and outpatient visits were also decreased [9].

The administration of the obstetrics and gynecology emergency department (OG-ED) likewise faces open challenges regarding overcrowding and all associated complications [10]. Nearly one-third of trips to the OG-ED are for non-urgent treatments. Therefore, during the COVID-19 pandemic, the renovation of emergency services could markedly modify access and hospitalizations in large OG-EDs [11].

The aim of this study was to analyze how the restrictions used during the lockdown period and subsequent months of 2020 affected the trends of the access of emergency services and hospitalization in a tertiary-care OG-ED, compared to the corresponding periods of the previous year.

## 2. Materials and Methods

We conducted a single-center retrospective analysis on emergency medical records including a wide time window from January to December 2020 in a tertiary-care university emergency section related to the Obstetrics and Gynecology Unit, AOU Luigi Vanvitelli, Naples, Italy.

According to the Italian pandemic waves of 2020, three different timeframes were obtained:

- January to February 2020 (before COVID-19 pandemic);
- March to June 2020 (nationwide lockdown period);
- July to September 2020 (removal of restrictive measures);
- October to December 2020 (regional lockdown according to red, yellow and green zones related to each region’s contagion curve) [12].

Each one of the above-mentioned time windows was compared to the corresponding period of 2019, before the spreading of COVID-19.

The Helsinki Declaration, the Committee on Publication Ethics (COPE) standards (http://publicationethics.org/, accessed on 7 October 2023), and the Reporting of studies Conducted using Observational Routinely collected health Data (RECORD) Statement are followed in the design, analysis, interpretation of data, writing, and revisions, available through the Enhancing the Quality and Transparency of Health Research (EQUATOR) network (www.equator-network.org, accessed on 7 October 2023). Given that the study was an observational one, the data obtained were anonymized to remove any information that may be used to formally identify the patients. Each participant in this study received information about the methods and provided their written agreement to enable data to be collected and analyzed for research.

The study included all the OG-ED admissions, classifying all pregnant patients as obstetric patients (from 0 to 22 gestational weeks, from 22 to 35 weeks, and from 35 weeks to term, whether single or multiparous women), as well as puerperal patients as gynecological patients. The study’s findings were included for all patients who used the OG-ED.

Patients with missing data from the centralized hospitalization system, patients who left the OG-ED before the final diagnosis, or that denied consent to acquire data were therefore excluded from the analysis.

The primary outcome was the evaluation of changes in OG-ED admissions between each 2020 timeframe related to restrictive COVID-19 measures compared to the same timeframes in 2019. The secondary outcome was the evaluation of changes in terms of color code access and hospitalizations between the aforementioned timeframes. Moreover, a descriptive analysis of reasons for OG-ED access and hospitalizations was carried out to better describe the clinical scenarios.

Gynecologists, midwives, anesthetists, neonatologists, social workers, and nurses are part of the multidisciplinary team at the OG-ED. Our OG-ED operates around the clock in conjunction with the radiology and laboratory departments. Professional midwives handle the triage. The on-call doctor then assesses the patients in accordance with the codes. The following categories apply to the administration of triage codes in the hospital protocol when the patient is admitted: The yellow code means “urgency”, which is the threat of impairment of a vital function of the woman or fetus at a gestational age of 23 weeks to be evaluated within 15 min; the red code means “emergency”, which is the current impairment of a vital function of the woman or fetus at a gestational age of 23 weeks; the green code denotes “non-urgency”, or services that may be delayed and reviewed within three hours; the white code denotes treatments that are comparable to outpatient care. The patient is discharged from the OG-ED with a discharge color when the final diagnosis has been determined, which is frequently different from the color that was allocated during triage. The color code assigned at the admission was used to evaluate the primary and secondary outcomes of the study.

Data were obtained and anonymously collected from the hospital management software for emergency access, hospitalization, and medical charts (Hero4 version 4.30, Dedalus Global S.p.A., Florence, Italy).

For each patient, the following data were collected: Month and year of the access; type of access (obstetric or gynecological); gestational age; cause of the access; OG-ED triage admission; hospitalization following OG-ED access. 

Because standard clinical procedures have been followed, the study did not need approval from an institutional or ethical review body since it was classified as retrospective chart analysis of routine clinical practice.

### Statistical Analysis

SPSS software (IBM Corp. Version 27.0, Armonk, NY, USA) was used for statistical analysis. Categorical data were analyzed by means of the chi-square test for independence, expanded Fisher exact test, and Bonferroni–Holms corrections for multiple testing for the primary and secondary outcomes, as appropriate. After the application of multiple testing corrections, a *p*-value (*p*) < 0.0125 was considered statistically significant.

## 3. Results

### 3.1. Primary Outcome: OG-ED Accesses

Overall, from January to December, 4233 accesses at the OG-ED were reported for 2019 while 3652 women were admitted in 2020, showing a decreasing trend of −13.7%

Table 1 shows that between March and June 2020, when the country was in total lockdown, the overall number of patients attending the OG-ED decreased (1124 in 2020 vs. 1477 in 2019). However, the analysis of differences among paired timeframes showed a marked reduction between March and June 2019–2020 with July–September and October–December timeframes (Δ −23.5% vs. −3.1% and −5.9%; *p* = 0.001, respectively) but not January–February 2019–2020 (Δ −23.5% vs. −18.5%; *p* = 0.356) (Table 1).

Similarly, there were no retrievable differences regarding the change in rates between obstetric and gynecological accesses among paired timeframes (Table 1).

### 3.2. Secondary Outcome: Changes in Color Code of Accesses

Table 2 summarizes data for the comparisons of OG-ED access color codes for the study period’s timeframes. 

While the number of green codes fell due to severe lockdown in March–June compared to other paired timeframes (Δ −12.0% vs. −8.4% and −4.1% from July–September and October–December, respectively; *p* = 0.001), a significant increase in yellow codes was observed in the same period (Δ +7.9% vs. +5.1% and +1.2% from July–September and October–December, respectively; *p* = 0.001) (Table 2). However, such trends were also seen in the January–February 2019 and 2020 timeframes (Δ −16.4%; *p* = 0.001 for green codes and +9.7%; *p* = 0.001 for yellow codes, respectively) (Table 2).

### 3.3. Descriptive Analysis of OG-ED Accesses

Gestational age at the OG-ED access with related reason for admission are reported in Table 3. 

### 3.4. Hospitalizations 

Table 4 shows how many patients evaluated in the OG-ED between March and December of 2019 and 2020 were hospitalized.

There was a significant increase in hospitalizations from October–December relative to March–June and July–September (Δ +4.6% vs. +2.4% and +2.5%, respectively; *p* = 0.001) 2019–2020.

They were mainly related to abdominal pain after 22 weeks, labor, non-stress tests, hypertension, diabetes, or other pregnancy complications (raised transaminases or bile acids) or syncope (Table 4).

## 4. Discussion

During the lockdown period (March to June 2020), most hospital facilities were overwhelmed by COVID-19 cases, making them inaccessible to non-COVID patients, including pregnant women, due to health restrictions imposed by the government, resulting in a decrease in OG-ED accesses of about 13% [13,14]. Although the suspension of elective admissions and outpatient services for high-risk pregnancies was expected to result in an increase in hospitalizations during the COVID-19 period [15,16], this retrospective study showed that the overall rate of emergency admissions slightly decreased in the evaluated timeframes [17] but had a declining trend that may have already been present in the pre-COVID-19 era [18].

To date, four studies have reported the influence of COVID-19 on OG-ED accesses in Italy [11,19,20,21]. All of them reported in favor of a significant impact on admissions and hospitalizations which could have deeply reduced the quality of health in pregnant and non-pregnant women. However, our study showed a non-significant decrease when, for the first time, a side-by-side comparison based on the different subtypes of restrictions between two consecutive years with and without COVID-19 restrictions was made. 

The study from Carbone et al. [21] reported data from the same Italian region as our research. They reported a significant reduction of both admissions and hospitalizations in each month of 2020 compared to 2019. Conversely, we reported that only the lockdown period (March to June 2020) was deemed to have a substantial impact on OG-ED admissions, while hospitalizations remained mainly untouched. The use of different admission policies could be one of the most compelling explanations. Differently from our institution, the hospital of Carbone et al. [21] was a referral center for COVID-19-positive pregnancies, which might also explain the different rates of admissions and hospitalizations. 

Amadori et al. [11] reported a reduction of more than the half for accesses from 2019 to 2020, which was similar to Grandi et al.’s [20] study results, which showed that there was a reduction in accesses and hospitalizations when comparing the timeframes 1–30 November 2019 and 11 March–9 April 2020. Similar results were also reported by Dell’Utri et al. [19] for the timeframes 23 February–23 June 2019 and 2020.

Conversely, we reported that the same trends of an overall decrease in OG-ED admissions and changes concerning the reduction of non-serious cases and the increase in those with higher priority were present even before the inclusion of the restraint measures due to COVID-19. In some situations, the reduction of cases marked as green codes was even greater than when the national lockdown was applied; in fact, we recorded a 16.4% reduction between January and February 2019–2020 compared to 12.0% of the March–June period of the same two years. This condition was also noted for the increase in yellow codes, with a greater increase even before the implementation of the restrictive measures. 

Multiple explanations could be attributed to these findings. Firstly, one reason may the increased fear of contagion related to media reports that may have played a conditioning role on public health even before the restrictions [22]. On the contrary, analyzing the data on admissions and hospitalizations in 2019 more deeply, our and other hospitals might show a decreasing trend that was already present, so the impact of restrictive measures on the total cost computation related to hospitalizations would be even less than what was expected. Therefore, this evidence needs further retrospective analyses to overcome previous limitations.

During the lockdown, we also noted increased gynecological hospitalizations resulting from pelvic abdominal pain, particularly acute adnexal pathologies, and oncological emergencies. This strengthens the evidence that the reduced outpatient and regular activities of Italian hospitals increased the need for urgent admissions and hospitalizations and that pain, rather than blood loss, is still reported as the most unpleasant and worrying symptom that pushes women to seek emergency care [23,24].

From July to September 2020, we could not observe changes in obstetric patients’ accesses. This was partially expected because during this period there was a reopening, albeit partial, of facilities and outpatient clinics, so that patients were freer both in terms of travel and access to services [25]. This suggests that while the national closure caused a large proportion of people to minimize travel for fear of infection, the introduction of regional lockdowns did not have a negative impact on hospital access, probably due to greater awareness of the nature and mode of infection [26,27,28].

Interestingly, hypertension and obstetrics women with gestational diabetes or cholestatic and hepatic issues were all increased in 2020 compared to 2019. With this finding, in accordance with Carbone et al. [21] and La Verde et al. [12], it was reasonable to suppose that the lifestyle restrictions of the lockdown would have raised the likelihood of the emergence of such issues. Indeed, a decrease in physical activity, an increase in house rest, an associated rise in maternal weight, as well as stress and worry, might all be potential contributors to the rise in blood pressure [29,30,31,32]. 

However, this study has several limitations. Possible flaws in the recording of data might have influenced the retrospective collection of patient data. In the computerized records, the final diagnoses were heterogeneously categorized, therefore their inclusion in a specific subcategory could be biased by the operator’s decisional process. Similarly, all the biases in its retrospective design (including selection bias) should be considered while reading the results. Concerning the admission to the OG-ED, although the triage midwives’ characterization of the primary complaint was often uniform, it is also conceivable that they had a varied interpretation of the patients’ concerns. It appears plausible to assume that this minor scenario may occur again in additional, related epidemiological scenarios [9].

Nonetheless, there are several points of strength that could be attributed to our analysis. First, the introduction of a dedicated pathway with universal antigen screening tests and the strict scheduling of maternity admissions with prior SARS-CoV-2 molecular swabs have helped to ensure trust and safety, preventing a reduction in emergency services in our emergency department and outpatient facilities. Moreover, the exclusion of patients with missing data was useful to avoid confounding. 

## 5. Conclusions

Our study did not show a marked reduction in the number of OG-ED admissions due to COVID-19, showing that OG-ED accesses were decreasing even before the application of national restrictions. Obstetric care seemed less affected by the pandemic, highlighting the importance of antenatal care and screening at all stages. However, there were differences between the pandemic and pre-pandemic periods in terms of presenting to the OG-ED. Although the suspension of elective admissions and outpatient services for high-risk pregnancies was expected to result in an increase of hospitalizations during the COVID-19 lockdown period, the only significant trend of increased hospitalizations was seen during the regional lockdown period (October–December 2020), probably due to reasons not related to the COVID-19 emergency. 

## Figures and Tables

**Table 1:** Accesses to the OG-ED: Comparison of 2019 and 2020.

	January–February 2019	January–February 2020	Δ (%)	March–June 2019	March–June 2020	Δ (%)	July–September 2019	July–September 2020	Δ (%)	October–December 2019	October–December 2020	Δ (%)	*p*-Value
Accesses to OG-ED	764	623	−18.5	1477	1124	−23.9 *	1079	1046	−3.1	913	859	−5.9	0.001
Obstetric accesses	649/764 (84.9%)	518/623 (83.1%)	−1.8	1258/1477 (85.2%)	946/1124 (84.2%)	−1.0	922/1079 (85.4%)	859/1046 (82.1%)	−2.3	759/913 (83.1%)	730/859 (85%)	+1.9	0.883
Gynecological accesses	115/764 (15.1%)	105/623 (16.9%)	+1.8	219/1477 (14.8%)	178/1124 (15.8%)	+1.0	157/1079 (14.6%)	187/1046 (17.9%)	+2.3	154/913 (16.9%)	129/859 (15%)	−1.9	

* *p* = 0.001 vs. July–September and October–December.

**Table 2:** Comparison of OG-ED accesses according to triage color codes for evaluated timeframes.

	January–February 2019	January–February 2020	Δ (%)	March–June 2019	March–June 2020	Δ (%)	July–September 2019	July–September 2020	Δ (%)	October–December 2019	October–December 2020	Δ (%)	*p*-Value
Color code													
White	68/764 (8.9%)	86/623 (13.8%)	+4.9	178/1477 (12.1%)	145/1124 (12.9%)	+0.8	143/1079 (13.3%)	159/1046 (15.2%)	+1.9	118/913 (12.9%)	130/859 (15.1%)	+2.2	0.004
Green	658/764 (86.1%)	434/623 (69.7%)	−16.4	1240/1477 (84%)	809/1124 (72%)	−12.0	810/1079 (75.1%)	698/1046 (66.7%)	−8.4	628/913 (68.8%)	556/859 (64.7%)	−4.1	0.001
Yellow	36/764 (4.7%)	90/623 (14.4%)	+9.7	53/1477 (3.5%)	128/1124 (11.4%)	+7.9	91/1079 (8.4%)	141/1046 (13.5%)	+5.1	129/913 (14.1%)	131/859 (15.3%)	+1.2	0.001
Red	2/764 (0.3%)	13/623 (2.1%)	+1.8	6/1477 (0.4%)	42/1124 (3.7%)	+3.3	35/1079 (3.2%)	48/1046 (4.6%)	+1.4	38/913 (4.2%)	42/859 (4.9%)	+0.7	0.213

**Table 3:** Descriptive analysis of accesses to OG-ED between 2019 and 2020.

	January–February 2019	January–February 2020	March–June 2019	March–June 2020	July–September 2019	July–September 2020	October–December 2019	October–December 2020
Gestational age at access (weeks)								
<22	248/649 (38.2%)	173/518 (33.4%)	429/1258 (34%)	271/946 (28.6%)	259/922 (28.1%)	250/859 (29.1%)	255/759 (33.6%)	184/730 (25.2%)
>22 and <35	91/649 (14.1%)	66/518 (12.7%)	187/1258 (15%)	124/946 (13.1%)	121/922 (13.1%)	119/859 (13.9%)	116/759 (15.3%)	103/730 (14.1%)
>35	304/649 (46.8%)	264/518 (51%)	634/1258 (50.4%)	540/946 (57.1%)	524/922 (56.8%)	482/859 (56.1%)	377/759 (49.7%)	436/730 (59.7%)
Reason								
Hyperemesis	11/649 (1.7%)	11/518 (2.1%)	15/1258 (1.2%)	2/946 (0.2%)	15/922 (1.6%)	12/859 (1.4%)	16/759 (2.1%)	1/730 (0.1%)
Metrorrhagia before the 22nd gestational week	68/649 (10.5%)	88/518 (17.1%)	98/1258 (7.8%)	136/946 (14.4%)	80/922 (8.7%)	109/859 (12.7%)	125/759 (16.5%)	100/730 (13.7%)
Pain before the 22nd gestational week	164/649 (25.3%)	60/518 (11.6%)	301/1258 (24%)	98/946 (10.4%)	151/922 (16.4%)	109/859 (12.7%)	93/759 (12.3%)	62/730 (8.5%)
Metrorrhagia after the 22nd gestational week	10/649 (1.5%)	10/518 (1.9%)	7/1258 (0.6%)	25/946 (2.6%)	20/922 (2.1%)	24/859 (2.8%)	31/759 (4.1%)	16/730 (2.2%)
Abdominal pain after the 22nd gestational week, labor, non-stress test	335/649 (51.6%)	218/518 (42.1%)	734/1258 (58.3%)	442/946 (46.7%)	496/922 (53.8%)	382/859 (44.5%)	306/759 (40.3%)	358/730 (49%)
Pre-labor rupture of the membranes	23/649 (3.5%)	42/518 (8.1%)	28/1258 (2.2%)	75/946 (7.9%)	58/922 (6.3%)	100/859 (11.6%)	69/759 (9.1%)	88/730 (12%)
Fetal growth restriction, reduction of fetal movements, oligohydramnios, polyhydramnios	1/649 (0.2%)	23/518 (4.4%)	7/1258 (0.6%)	26/946 (2.7%)	17/922 (1.8%)	22/859 (2.6%)	28/759 (3.7%)	19/730 (2.6%)
Post-term pregnancy/scheduled hospitalization	7/649 (1.1%)	10/518 (1.9%)	16/1258 (1.2%)	41/946 (4.3%)	27/922 (2.9%)	21/859 (2.4%)	20/759 (2.6%)	26/730 (3.6%)
Hypertension, diabetes or cholestatic/hepatic issues (raised transaminases and/or bile acids), syncope	6/649 (0.9%)	15/518 (2.9%)	8/1258 (0.6%)	49/946 (5.1%)	10/922 (1.1%)	38/859 (4.4%)	16/759 (2.1%)	32/730 (4.4%)
Post-discharge complications	6/649 (0.9%)	15/518 (2.9%)	8/1258 (0.6%)	11/946 (1.2%)	18/922 (2%)	8/859 (0.9%)	11/759 (1.4%)	7/730 (1%)
Others	18/649 (2.8%)	26/518 (5%)	36/1258 (2.9%)	43/946 (4.5%)	30/922 (3.3%)	34/859 (4%)	44/759 (5.8%)	21/730 (2.9%)

**Table 4:** Hospital admissions from OG-ED: comparison of results for 2019 and 2020.

	March–June 2019		March–June 2020		July–September 2019		July–September 2020		October–December 2019		October–December 2020		*p*-Value
Hospitalization	Number/Accesses	%	Number/Accesses	%	Number/ Accesses	%	Number/Accesses	%	Number/Accesses	%	Number/ Accesses	%	
	300/1250	24.0	239/904	26.4	277/851	32.5	328/935	35.0	252/748	33.7	277/723	38.3	0.001
Gestational age at hospitalization (weeks)													
<22	62/429	14.4	36/259	13.8	40/250	16.0	58/271	21.1	47/255	18.4	40/184	21.7	0.519
>22 and <35	17/187	9	14/121	11.6	18/119	15.1	18/124	14.5	12/116	10.3	18/103	17.4	
>35	221/634	35	189/524	36.1	219/482	45.4	252/540	46.6	193/377	51.2	219/436	50.2	
Reason for obstetric hospitalization													
Metrorrhagia before the 22 GW	25/98	25.5	23/80	28.7	31/109	28.4	43/136	31.6	35/125	28.0	31/100	31.0	<0.001
Pain before the 22 GW	32/301	10.6	7/151	4.6	2/109	1.2	3/98	3.0	6/93	6.4	2/62	3.2	
Metrorrhagia after the 22 GW	2/7	28.6	3/20	15.0	1/24	4.2	1/25	4.0	6/31	19.3	1/16	6.2	
Abdominal pain after the 22 GW, labor, non-stress test	194/734	26.4	119/496	24.0	121/382	31.7	139/442	31.4	107/306	34.9	121/358	33.8	
Fetal growth restriction, reduction of fetal moves, oligohydramnios, polyhydramnios	1/7	14.3	6/17	35.3	12/22	54.5	14/26	53.8	8/28	28.6	12/19	63.1	
Hypertension, diabetes or cholestatic/hepatic issues (raised transaminases and/or bile acids), syncope	5/8	62.5	9/10	90.0	18/38	47.4	24/49	49.0	11/21	52.4	18/32	56.2	
Reason for gynecological hospitalization													
Pelvic abdominal pain (also scheduled admissions for cysts, prolapse, renal colic, ovarian carcinoma, myoma)	12/137	8.7	8/70	11.4	12/60	20.0	14/68	20.6	6/48	12.5	12/58	20.7	0.001
Menometrorrhagia (including cervical and endometrial cancer)	4/33	12.1	7/41	17.1	12/66	18.2	10/57	17.5	9/48	18.8	12/48	25.0	

GW: gestational weeks.

## Data Availability

Data and material are available from the corresponding author upon reasonable request.

## References

[1] Porto E.D., Naticchioni P., Scrutinio V. (2022). Lockdown, essential sectors, and COVID-19: Lessons from Italy. J. Health Econ..

[2] Sebastiani G., Palu G. (2020). COVID-19 and School Activities in Italy. Viruses.

[3] Bressy S., Zingarelli E.M. (2022). COVID-19 and primary care in Italy: One year later. J. Prim. Health Care.

[4] Chiesa R., Kahlberg A., Rinaldi E., Mascia D. (2021). Emergency management of the COVID-19 pandemic in a vascular surgery department of a large metropolitan hospital in Italy. Preparation, escalation, de-escalation, and normal activity. J. Card. Surg..

[5] Eichenberg C., Grossfurthner M., Kietaibl S., Riboli G., Borlimi R., Holocher-Benetka S. (2021). Emotional distress in the early stages of the COVID-19 related lockdowns depending on the severity of the pandemic and emergency measures: A comparative online-survey in Germany, Austria and Italy. BMC Psychiatry.

[6] Giorgi P.D., Gallazzi E., Capitani P., D’Aliberti G.A., Bove F., Chiara O., Peretti G., Schiro G.R. (2020). How we managed elective, urgent, and emergency orthopedic surgery during the COVID-19 pandemic: The Milan metropolitan area experience. Bone Jt. Open.

[7] Vitale S.G., Carugno J., Riemma G., Farkas Z., Krasznai Z., Bacsko G., Lampe R., Torok P. (2020). The role of hysteroscopy during COVID-19 outbreak: Safeguarding lives and saving resources. Int. J. Gynaecol. Obstet..

[8] Bongiovanni M., Arienti R., Bini F., Bodini B.D., Corbetta E., Gianturco L. (2021). Differences between the waves in Northern Italy: How the characteristics and the outcome of COVID-19 infected patients admitted to the emergency room have changed. J. Infect..

[9] Capanna F., Haydar A., McCarey C., Bernini Carri E., Bartha Rasero J., Tsibizova V., Helmer H., Makatsarya A., Di Renzo G.C. (2022). Preparing an obstetric unit in the heart of the epidemic strike of COVID-19: Quick reorganization tips. J. Matern. Fetal Neonatal Med..

[10] Villar J., Ariff S., Gunier R.B., Thiruvengadam R., Rauch S., Kholin A., Roggero P., Prefumo F., do Vale M.S., Cardona-Perez J.A. (2021). Maternal and Neonatal Morbidity and Mortality Among Pregnant Women with and without COVID-19 Infection: The INTERCOVID Multinational Cohort Study. JAMA Pediatr..

[11] Amadori R., Buscemi R., Desando A., Grillo F., Remorgida V., Surico D. (2022). Obstetrics and Gynecology Emergency Department Activity during Lockdown in a Teaching Hospital, Hub Center, for COVID-19. Obstet. Gynecol. Int..

[12] La Verde M., Torella M., Riemma G., Narciso G., Iavarone I., Gliubizzi L., Palma M., Morlando M., Colacurci N., De Franciscis P. (2022). Incidence of gestational diabetes mellitus before and after the COVID-19 lockdown: A retrospective cohort study. J. Obstet. Gynaecol. Res..

[13] Athiel Y., Civadier M.S., Luton D., Ceccaldi P.F., Bourret A., Sroussi J., Mandelbrot L., Ville Y., Nizard J., Sibony O. (2020). Impact of the outbreak of SARS-CoV-2 infection on urgent gynecological care. J. Gynecol. Obstet. Hum. Reprod..

[14] Wolf T.G., Schulze R.K.W., Ramos-Gomez F., Campus G. (2022). Effectiveness of Telemedicine and Teledentistry after the COVID-19 Pandemic. Int. J. Environ. Res. Public Health.

[15] Ciarleglio F.A., Rigoni M., Mereu L., Tommaso C., Carrara A., Malossini G., Tateo S., Tirone G., Bjerklund Johansen T.E., Benetollo P.P. (2021). The negative effects of COVID-19 and national lockdown on emergency surgery morbidity due to delayed access. World J. Emerg. Surg..

[16] Dashraath P., Wong J.L.J., Lim M.X.K., Lim L.M., Li S., Biswas A., Choolani M., Mattar C., Su L.L. (2020). Coronavirus disease 2019 (COVID-19) pandemic and pregnancy. Am. J. Obstet. Gynecol..

[17] Maranto M., Zaami S., Restivo V., Termini D., Gangemi A., Tumminello M., Culmone S., Billone V., Cucinella G., Gullo G. (2023). Symptomatic COVID-19 in Pregnancy: Hospital Cohort Data between May 2020 and April 2021, Risk Factors and Medicolegal Implications. Diagnostics.

[18] Santi L., Golinelli D., Tampieri A., Farina G., Greco M., Rosa S., Beleffi M., Biavati B., Campinoti F., Guerrini S. (2021). Non-COVID-19 patients in times of pandemic: Emergency department visits, hospitalizations and cause-specific mortality in Northern Italy. PLoS ONE.

[19] Dell’Utri C., Manzoni E., Cipriani S., Spizzico C., Dell’Acqua A., Barbara G., Parazzini F., Kustermann A. (2020). Effects of SARS CoV-2 epidemic on the obstetrical and gynecological emergency service accesses. What happened and what shall we expect now?. Eur. J. Obstet. Gynecol. Reprod. Biol..

[20] Grandi G., Del Savio M.C., Caroli M., Capobianco G., Dessole F., Tupponi G., Petrillo M., Succu C., Paoletti A.M., Facchinetti F. (2020). The impact of COVID-19 lockdown on admission to gynecological emergency departments: Results from a multicenter Italian study. Int. J. Gynaecol. Obstet..

[21] Carbone L., Raffone A., Travaglino A., Sarno L., Conforti A., Gabrielli O., De Vivo V., De Rosa M., Migliorini S., Saccone G. (2022). Obstetric A&E unit admission and hospitalization for obstetrical management during COVID-19 pandemic in a third-level hospital of southern Italy. Arch. Gynecol. Obstet..

[22] Meller F.O., Schafer A.A., Quadra M.R., Demenech L.M., Paludo S.D.S., da Silva P.A., Neiva-Silva L., Dumith S.C. (2022). Fear of COVID-19 and health-related outcomes: Results from two Brazilian population-based studies. Psychiatry Res..

[23] Alfieri N., Manodoro S., Marconi A.M. (2020). COVID-19 does not stop obstetrics: What we need to change to go on safely birthing. The experience of a University Obstetrics and Gynecology Department in Milan. J. Perinat. Med..

[24] Fumagalli S., Borrelli S., Ornaghi S., Vergani P., Nespoli A. (2023). Midwives’ experiences of providing maternity care to women and families during the COVID-19 pandemic in Northern Italy. Women Birth.

[25] Miralles O., Sanchez-Rodriguez D., Marco E., Annweiler C., Baztan A., Betancor E., Cambra A., Cesari M., Fontecha B.J., Gasowski J. (2021). Unmet needs, health policies, and actions during the COVID-19 pandemic: A report from six European countries. Eur. Geriatr. Med..

[26] Geremia S., Valente E.P., Mariani I., Dalena P., Lazzerini M. (2023). Women’s suggestions on how to improve the quality of maternal and newborn care during the COVID-19 pandemic in Italy: A co-occurrence network analysis. J. Glob. Health.

[27] McElfish P.A., Willis D.E., Shah S.K., Bryant-Moore K., Rojo M.O., Selig J.P. (2021). Sociodemographic Determinants of COVID-19 Vaccine Hesitancy, Fear of Infection, and Protection Self-Efficacy. J. Prim. Care Community Health.

[28] Heiat M., Heiat F., Halaji M., Ranjbar R., Tavangar Marvasti Z., Yaali-Jahromi E., Azizi M.M., Morteza Hosseini S., Badri T. (2021). Phobia and Fear of COVID-19: Origins, complications and management, a narrative review. Ann. Ig. Med. Prev. Comunità.

[29] Hori N., Shiraishi M., Harada R., Kurashima Y. (2021). Association of Lifestyle Changes Due to the COVID-19 Pandemic with Nutrient Intake and Physical Activity Levels during Pregnancy in Japan. Nutrients.

[30] Gow M.L., Rossiter C., Roberts L., Henderson M.J., Yang L., Roche J., Hayes E., Canty A., Denney-Wilson E., Henry A. (2022). COVID-19, lifestyle behaviors and mental health: A mixed methods study of women 6 months following a hypertensive pregnancy. Front. Public Health.

[31] Scott J., Hauspurg A., Davis E.M., Bryan S., Catov J.M. (2023). Maternal Mental Health, COVID-19-Related Distress, and Disruptions in Lifestyle Behaviors among Postpartum Mothers with a Previous Hypertensive Disorder of Pregnancy. J. Cardiovasc. Nurs..

[32] Roggio F., Trovato B., Ravalli S., Di Rosa M., Maugeri G., Bianco A., Palma A., Musumeci G. (2021). One Year of COVID-19 Pandemic in Italy: Effect of Sedentary Behavior on Physical Activity Levels and Musculoskeletal Pain among University Students. Int. J. Environ. Res. Public Health.

